# Release of extracellular ATP by bacteria during growth

**DOI:** 10.1186/1471-2180-13-301

**Published:** 2013-12-24

**Authors:** Roberto Mempin, Helen Tran, Connie Chen, Hao Gong, Katharina Kim Ho, Sangwei Lu

**Affiliations:** 1 Division of Infectious Diseases and Vaccinology, School of Public Health, University of California, Berkeley, CA 94720-7354, USA; 2 Current address: Technische Universität Dresden, Dresden, Germany

**Keywords:** ATP, Secretion, Bacteria, *Salmonella*, *E. coli*, Stationary survival

## Abstract

**Background:**

Adenosine triphosphate (ATP) is used as an intracellular energy source by all living organisms. It plays a central role in the respiration and metabolism, and is the most important energy supplier in many enzymatic reactions. Its critical role as the energy storage molecule makes it extremely valuable to all cells.

**Results:**

We report here the detection of extracellular ATP in the cultures of a variety of bacterial species. The levels of the extracellular ATP in bacterial cultures peaked around the end of the log phase and decreased in the stationary phase of growth. Extracellular ATP levels were dependent on the cellular respiration as bacterial mutants lacking cytochrome bo oxidase displayed lower extracellular ATP levels. We have also shown that *Escherichia coli* (*E. coli*) and *Salmonella* actively depleted extracellular ATP and an ATP supplement in culture media enhanced the stationary survival of *E. coli* and *Salmonella*. In addition to *E. coli* and *Salmonella* the presence of the extracellular ATP was observed in a variety of bacterial species that contain human pathogens such as *Acinetobacter*, *Pseudomonas*, *Klebsiella* and *Staphylococcus*.

**Conclusion:**

Our results indicate that extracellular ATP is produced by many bacterial species during growth and extracellular ATP may serve a role in the bacterial physiology.

## Background

Adenosine triphosphate (ATP) is one of the most important small molecules in the living organisms. It is a universal energy currency used in many biological processes that require energy. Living organisms generate ATP through respiration and subsequently utilize ATP to carry out cellular functions that are necessary for their survival, growth and replication. In addition to its intracellular roles in storing and supplying energy in metabolism and enzymatic reactions, ATP also has signaling functions. ATP has been shown to control the differentiation of T_H_17 cells in intestinal lamina propria [1]. Extracellular ATP has been shown to interact with P2 receptors to modulate immune response by stimulating cell migration and cytokine secretion (reviewed in [2,3]). Recently, ATP was also shown to regulate virulence gene *mgtC* in *Salmonella*[4]. These findings suggest that ATP is a more versatile molecule than a supplier of energy in both prokaryotic and eukaryotic organisms.

ATP is present in all living organisms, consistent with its roles in biological reactions and processes. The intracellular ATP level in *Escherichia coli* (*E. coli*) and *Salmonella* is reported to be 1–5 mM and changes according to various environmental and physiological conditions of bacteria [5-8]. A few reports in recent years described the detection of the extracellular ATP from selected bacterial species [9-11]. Iwase et al. reported that ATP was detected at 1–3 μM from the supernatant of the stationary cultures of *Enterococcus gallinarum* isolated from mouse and human feces, but not from the *E. coli* and *Staphylococcus aureus* strains tested in the same study [10]. In a follow-up study published recently the same group reported that ATP release is dependent on glycolysis [11]. An earlier report by Ivanova et al. showed that bacteria from a variety of genera including *Sulfitobacter*, *Staleya* and *Marinobacter* release ATP to concentrations ranging from 0.1 pM to 9.8 pM/colony forming unit (CFU) or 190 μM to 1.9 mM [9]. The purpose and significance of the ATP release is currently unknown. A possible role for the extracellular ATP in the intestines was proposed to be activating a unique subset of lamina propria cells, CD70^high^CD11c^low^ cells, which facilitates the differentiation of T_H_17 cells [1]. Consistent with the hypothesis, ATP release was detected from commensal bacteria (and to a lesser extent from *Salmonella*) cultured *in vitro* which may explain the source of the intestinal ATP [1].

In this study, we examined if extracellular ATP is present in the culture supernatant of Gram-negative bacteria *E. coli* and *Salmonella* and investigated if extracellular ATP has biological functions in bacterial growth and survival. Here we report that extracellular ATP can be detected transiently in cultures of Gram-negative bacteria *E. coli* and *Salmonella*. In addition, we show that extracellular ATP can also be detected in clinical isolates of many bacterial species suggesting that ATP release is not limited to selected bacterial species.

## Methods

### Bacterial strains, bacterial cultures and reagents

*Acinetobacter*, *Klebsiella* and *Pseudomonas* strains used in the study are clinical isolates obtained from California State Department of Health. *Staphylococcus* strains were from American Type Culture Collection (Manassas, VA). Strains of *Salmonella enterica* Serovar Enteritidis and Typhimurium were clinical isolates and have been reported previously [12,13]. The clinical isolates were from routine surveillance efforts at California State Department of Health with no patient information and their handling conforms to the ethical requirements of University of California and National Institute of Health. *E. coli* K12 MG1655 was a gift from Dr. Sydney Kustu at the University of California, Berkeley (Table 1). *E. coli* mutants and the parental strain BW25113 were obtained from Coli Genetic Stock Center, Yale University (New Haven, CT) (Table 2).

**Table 1:** Bacterial strains used

**Bacterial strain**		**Characteristics**	**Source or reference**
*E. coli*			
	MG1655	*E. coli* K12 derivative	Dr. Sydney Kustu, UC Berkeley
	BW25113	*E. coli* K12 derivative	Coli Genetic Stock Center, Yale University
	KSU-9	Animal isolate of *E. coli* O157:H7	[12]
	KSU-12	Animal isolate of *E. coli* O157:H7	[12]
*Salmonella enterica* Serovar Enteritidis			
	SE2472	Clinical isolate, Phage type 4	[13]
	SE6782	Clinical isolate, Phage type 4	[13]
	SE8464	Clinical isolate, Phage type 4	[13]
	SE8743	Clinical isolate, Phage type 4	[13]
	SE10871	Clinical isolate, Phage type 4	[13]
	SE4052	Clinical isolate, Phage type 8	[13]
	SE4081	Clinical isolate, Phage type 8	[13]
	SE4191	Clinical isolate, Phage type 8	[13]
	SE4241	Clinical isolate, Phage type 8	[13]
	SE4386	Clinical isolate, Phage type 8	[13]
	SE2107	Clinical isolate, Phage type unknown	[12]
	SE2606	Clinical isolate, Phage type 8	[12]
	SE0052	Clinical isolate, Phage type 13	[12]
	SE0718	Clinical isolate, Phage type 4	[12]
	SE0430	Clinical isolate, Phage type 4	[12]
*Salmonella enterica* Serovar Typhimurium			
	ST3665	Clinical isolate	[12]
	ST3744	Clinical isolate	[12]
	ST3964	Clinical isolate	[12]
	ST3864	Clinical isolate	[12]
	ST10428	Clinical isolate	[12]
	ST2258	Clinical isolate	[12]
	ST2297	Clinical isolate	[12]
	ST2298	Clinical isolate	[12]
	ST2302	Clinical isolate	[12]
	ST2327	Clinical isolate	[12]
*Acinetobacter junii*			
	AJ4970	Clinical isolate	This study
	AJ4978	Clinical isolate	This study
*Pseudomonas aeruginosa*			
	PA292	Clinical isolate, CDPH*	This study
	PA4553	Clinical isolate, CDPH*	This study
*Klebsiella pneumonia*			
	KP2320	Clinical isolate, CDPH*	This study
	KP7690	Clinical isolate, CDPH*	This study
*Klebsiella oxytoca*			
	KO76	Clinical isolate, CDPH*	This study
*Staphylococus aureus*			
	SA25923	Clinical isolate	ATCC
	MRSA43300	Clinical isolate	ATCC

* CDPH, California Department of Public Health.

**Table 2:** ***E. coli *** **and *Salmonella *** **mutant strains**

***Salmonella enterica *** **Serovar Enteritidis**		
**Mutant**	**Characteristics**	**Source or reference**
Δ*cyoA*	SE2472 Δ*cyoA::kan*	This study
Δ*cyoB*	SE2472 Δ*cyoB::kan*	This study
Δ*cyoCD*	SE2472 Δ*cyoCD::kan*	This study
*E. coli* (from Coli Genetic Stock center)		
**Strain/mutant**	**Strain number**	**Source or reference**
BW25113 (wild type)	CGSC#: 7636	[19]
*∆appC*	JW0960-1	[19]
*∆cydB*	JW0723-2	[19]
*∆cyoA*	JW0422-1	[19]
*∆cyoC*	JW0420-1	[19]
*∆cyoD*	JW0419-1	[19]

Culture media Luria Bertani (LB) broth and M9 minimal medium were from BD Diagnostics (Sparks, MD). All bacteria were cultured in LB broth at 37°C with shaking at 225 rpm or as indicated. Bacterial culture density was measured by OD_600nm_ or by plating serially diluted cultures on LB agar plates and counting colonies after overnight incubation.

All chemical reagents were from Sigma Aldrich unless otherwise specified. BacTiter-Glo™ Microbial Cell Viability Assay Reagent was from Promega (Madison, WI).

### Determination of ATP level in bacterial culture

Bacteria were cultured in LB broth at 37°C overnight with shaking at 225 rpm. Overnight cultures were diluted 1:100 in fresh LB broth and cultured at 37°C with shaking. Aliquots of cultures were taken after 3, 6, 9, and 24 hours of incubation, and OD_600 nm_ was measured at each time point. Bacterial cultures were then centrifuged at 16,100 × *g* for 5 min. Culture supernatant was transferred to a fresh tube and stored at −80°C until assayed.

ATP level in bacterial supernatant was determined using BacTiter-Glo™ Microbial Cell Viability Assay Reagent (Promega, Madison, WI). It is a luciferase – based assay and the ATP level is determined by measuring luminescence levels and comparing to an ATP standard curve. One hundred microliters of culture supernatant were mixed with an equal volume of BacTiter-Glo™ Microbial Cell Viability Assay Reagent in a 96-well opaque plate and incubated at room temperature for 5 min. After incubation, luminescence was read in a SpectraMax M2 plate reader (Molecular Devices, Sunnyvale, CA). ATP standard solutions were prepared using adenosine 5-triphosphate disodium salt hydrate (A2383, Sigma Aldrich, St. Louis, MO) and a standard curve using 10-fold dilutions of ATP standard solutions prepared in H_2_O was included in each experiment. ATP concentrations in bacterial samples were determined by comparing to the ATP standard curve for each assay. LB, H_2_O or buffer was included in all assays as the negative controls.

The ATP level in bacterial cells was determined similarly as described for the culture supernatant. Bacteria were cultured in LB broth with shaking at 37°C. After various culture periods, an aliquot of a culture was collected for measuring OD_600nm_ and for preparing bacterial extracts using the perchloric acid extraction method [14]. Two hundred microliters of bacterial culture were mixed with 100 μl of ice – cold 1.2 M perchloric acid and vortexed for 10 seconds. The mixture was incubated on ice for 15 min. and spun down at 16,100 × *g* for 5 min. at 4°C. Two hundred microliters of supernatant were transferred to a fresh tube and mixed with 100 μl of a neutralizing solution containing 0.72 M KOH and 0.16 M KHCO_3_. The neutralized extract was then spun down at 16,100 × *g* for 5 min. and the supernatant was transferred to a fresh tube for use for theATP assay.

### ATP depletion Assay

Overnight cultures of bacteria were adjusted to OD_600nm_ = 3.0 and 1 mL of bacterial culture was spun down. The culture supernatant was transferred to a fresh tube and bacterial pellet was resupended in 1 ml of fresh LB. ATP was added to the culture supernatant or to the resuspended bacterial cells to 10 μM. All samples were incubated at 37°C. Aliquots of samples were collected after various time periods to determine ATP depletion by culture supernatant or by bacteria cells. ATP depletion by culture supernatant was determined by assaying the residual ATP level in the samples. ATP depletion by bacteria cells was determined by first spinning down bacterial culture to remove bacteria and then determining the residual ATP level in the culture supernatant. ATP depletion by killed bacteria was determined by first heating bacterial culture at 65°C for 20 min. before being used for the ATP depletion assay as described above for bacteria cells. A sample of LB broth supplemented with 10 μM ATP was included as a control in all assays to establish the stability of ATP in the LB broth.

ATP depletion of bacteria was also evaluated using ^35^S – or ^32^P – labeled ATP. Overnight cultures of bacteria were spun down and resuspended in equal volumes of LB supplemented with 10 nM of ^35^S-α-ATP or ^32^P -γ-ATP (1:1,000 dilution) (PerkinElmer, Waltham, MA). Aliquots of bacterial cultures were collected after various incubation periods and spun down, and the culture supernatant was transferred to a fresh tube. The bacterial pellet was then washed three times with PBS, resuspended in SOLVABLE aqueous – based solubilizer (PerkinElmer, Waltham, MA) and lysed at 65 C for 2 hours. Bacterial lysates were centrifuged at 16,100 × *g* for 5 min. and the cleared lysate was transferred to a fresh tube. Radioactivity levels in both culture supernatant and bacterial lysates were measured on a DELTA 300 model 6891 liquid scintillation system (TM Analytic, Inc.). Relative radioactivity in the culture supernatant and bacterial cells was calculated for each sample.

### Stationary phase survival

Overnight cultures of bacteria were diluted 1:100 in M9 minimal medium and cultured at 37°C with shaking for 7 days. In cultures with ATP supplement, ATP was added to desired concentration each day from day 2 to day 6. At day 2 and day 7 of incubation, an aliquot of bacterial culture was collected and the bacterial concentration (CFU/mL) was determined by plating. The survival of bacteria was measured by the ratio of bacterial CFU/mL at day 7 to that at day 2.

### Mutagenesis of *cyo* genes

The ∆*cyo* mutants of *Salmonella* were generated using the one-step mutagenesis procedure as described previously [15,16]. Primers used for mutagenesis are listed in Table 3. Primer pairs cyoA5KO-cyoA3KO, cyoB5KO-cyoB3KO and cyoC5KO-cyoD3KO were used to amplify a kanamycin resistance cassette (Kan^R^) from plasmid pKD4 by PCR and the PCR products were used for generating the ∆*cyoA*, ∆*cyoB* and ∆*cyoCD* mutants, respectively [15]. In ∆*cyoA* and ∆*cyoB* mutants, the *cyoA* or *cyoB* coding sequence was deleted and replaced with the Kan^R^. In the ∆*cyoCD* mutant, both *cyoC* and *cyoD* were deleted and replaced with Kan^R^ since the open reading frames of *cyoC* and *cyoD* overlap with each other. Each mutant was confirmed with flanking primers (cyoA5 and cyoA3, cyoB5 and cyoB3, or cyoCD5 and cyoCD3, respectively) and internal primers K1 and K2 in the Kan^R^ cassette (Table 3). Each mutation was transduced into fresh *Salmonella* by P22 transduction before being used for further analysis [17].

**Table 3:** Oligo nucleotides used for mutagenesis

**Oligonucleotides**	**Used for**	**Sequence**
cyoA5KO	Mutagenesis of *cyoA*	5′-ctcaggaaatacaataaaagtttgggatggttgtcattaattgcaggcactgcattactcagtggct gtaattctgcgctgctggatcccgtgtaggctggagctgcttc-3′
cyoA3KO	Mutagenesis of *cyoA*	5′-caacccttggagttggcggattccgcgtggctcatgtccataccttccattccttcatgcgagctgtgc tcaccttcaggttgggtcatgcatatgaatatcctccttag-3′
cyoB5KO	Mutagenesis of *cyoB*	5′-ataataagcaatcgttgcctgcgattaccctcgcagctattggggttgtctacggtgatattggt accagcccgctttatacgcttcgtgaatgtttgtcgtgtaggctggagctgcttc-3′
cyoB3KO	Mutagenesis of *cyoB*	5′-aaattatcactggatgcagtaccgttccatgaacctatcgtcatggtaacgatcgctgcaattatcgtc gggggactggcgatactggcagtgtaggctggagctgcttc -3′
cyoC5KO	Mutagenesis of *cyoC/D* mutation	5′-tgcattctgttctctattctgtttgctacctatgccgttctggtgaacggcaccgctggcggcccgacaggt aaggacattttcgaactggtgtaggctggagctgcttc -3′
cyoD3KO	Mutagenesis of *cyoC/D* mutation	5′-tgtagttgaggttccacataatccagatggagcccacaaccaggatggcgatgatcagcacggtaaag atgaaggccgtcatgttccagcatatgaatatcctccttag -3′
cyoA5	Confirmation of *cyoA* mutation	5′-atcatgtttacagtaatgta-3′
cyoA3	Confirmation of *cyoA* mutation	5′-tccgaacatcttatcttcct -3′
cyoB5	Confirmation of *cyoB* mutation	5′-aggaagataagatgttcgga-3′
cyoB3	Confirmation of *cyoB* mutation	5′-tcgcgtgcgttaaagtatca -3′
cyoCD5	Confirmation of *cyoC/D* mutation	5′-tgatactttaacgcacgcga -3′
cyoCD3	Confirmation of *cyoC/D* mutation	5′-tgcaggtattgcttaaacat-3′
K1	Kan^R^ primer for confirmation of mutation	5′-cagtcatagccgaatagcct-3′
K2	Kan^R^ primer for confirmation of mutation	5′-cggtgccctgaatgaactgca-3′

## Results

### ATP was detected in the culture supernatant of Gram-negative bacteria *Salmonella* and *E. coli*

**Figure 1:**
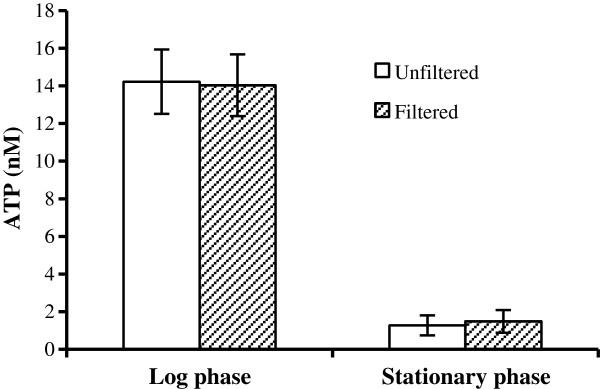
**ATP is present in the bacterial culture supernatant and the extracellular ATP is not due to bacteria contamination.** Overnight culture of *Salmonella* strain SE2472 was diluted 1:100 in LB and cultured at 37°C for 3 hours with shaking to reach early log phase. The overnight (stationary) and 3 hour (early log phase) cultures were spun down. An aliquot of each culture supernatant was filtered through a 0.22 μm filter to remove any residual bacteria. ATP levels in the filtered (hatched bars) or unfiltered culture supernatant (open bars) were measured. Results are the average of 3 assays and error bars represent standard deviations.

During a study on the role of bacterial physiological properties in the Type III secretion of *Salmonella,* we carried out experiments to measure the ATP levels in bacterial cells and used the culture supernatant as a negative control. Some culture supernatant samples unexpectedly displayed readily detectable signals in the ATP assay. We proceeded to determine if the ATP in the culture supernatant was due to a bacterial contamination of the culture supernatant. *Salmonella* cultures were grown at 37°C for 3 hours to the early log phase or overnight to the stationary phase and the cultures were spun down. The culture supernatant from each sample was transferred to a fresh tube and an aliquot was filtered through a 0.22 μm filter. ATP levels were determined in both filtered and unfiltered supernatant of the same culture and results were compared. ATP was detected in the supernatant of both early log and stationary phase cultures and filtration did not reduce the ATP levels (Figure 1). The ATP level in the supernatant of the stationary phase culture was just above the detection level (at approximately 1 nM), while the ATP level in the supernatant from the early log phase culture was noticeably higher at over 10 nM (Figure 1).

**Figure 2:**
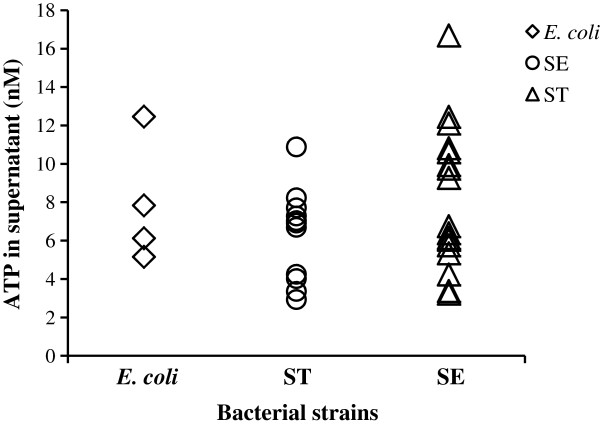
**ATP is present in the culture media of clinical and laboratory strains of *E. coli *and *Salmonella *during growth.** Overnight culture of each isolate was diluted 1:100 in fresh LB and cultured at 37°C with shaking. Early log phase bacterial cultures were harvested at 3 hours of incubation and ATP assays were carried out with culture supernatant. The ATP concentration was plotted for each bacterial isolate of *E. coli*, *Salmonella enterica* Serovar Enteritidis (SE) or *Salmonella enterica* Serovar Typhimurium (ST). The experiment was performed three times and results are from a representative experiment.

Next we tested if the extracellular ATP is only present in specific strains of *Salmonella* such as the clinical isolate SE2472 we used in the initial analysis. We tested a collection of clinical strains of *Salmonella* serovar Enteritidis (11 isolates) and Typhimurium (17 isolates), laboratory strains of *E. coli* K12 MG1655 and BW25113, and clinical strains of *E. coli* O157:H7 (2 isolates) (Table 1). Overnight culture of each bacterial strain was diluted 1:100 in fresh LB broth and cultured for 3 hours at 37°C with shaking. The ATP level in the culture supernatant was determined (Figure 2). The results showed that various bacterial strains displayed different levels of ATP in the culture supernatant; nevertheless extracellular ATP was detected in all isolates (Figure 2). These results raised a possibility that extracellular ATP is indeed present in the culture supernatant during growth.

### ATP level in the culture supernatant is regulated by growth phase

**Figure 3:**
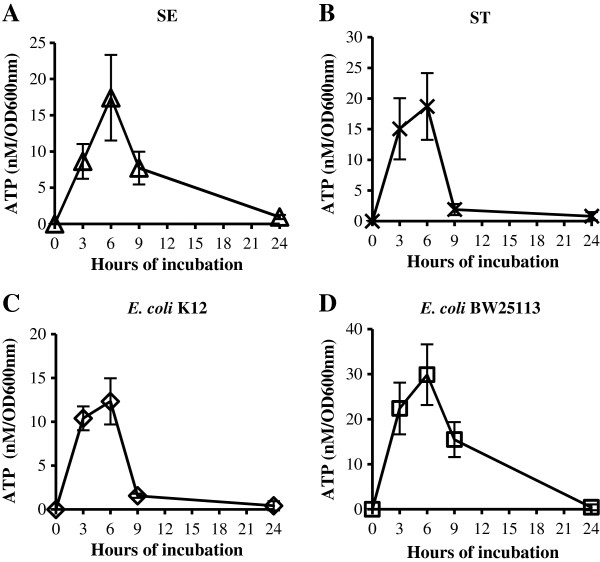
**Extracellular ATP level changes during bacteria growth.** Overnight cultures of *Salmonella* SE2472 **(A)** or ST14028s **(B)**, *E. coli* K12 **(C)** or BW25113 **(D)**, were diluted 1:100 in LB broth and cultured at 37°C with shaking. Aliquots were collected at various time points for measuring OD_600nm_ and culture supernatant was harvested for ATP assays. The ATP levels in the culture supernatant were normalized against OD_600nm_ and plotted against incubation period. Results are the average from 3 to 8 experiments and error bars represent standard deviations.

Since we detected a higher ATP level in the culture supernatant of the log phase cultures than that of the stationary phase cultures (Figure 1), we next investigated systematically if the ATP level in the culture supernatant changes during bacterial growth. Four bacterial strains were used for the analysis: *E. coli* K12 MG1655, *E. coli* K12 BW25113, *Salmonella enterica* Serovar Enteritidis SE2472 and *Salmonella enterica* Serovar Typhimurium ST14028s (Table 1). For each strain, an overnight culture of bacteria was diluted 1:100 in fresh LB broth and cultured at 37°C with shaking. Aliquots were taken at various time points to measure the bacterial density at OD_600nm_ and to determine the ATP level in the culture supernatant. The ATP level in the culture supernatant was normalized against OD_600nm_ and plotted against the incubation time for each strain (Figure 3). All strains displayed a bell – shaped curve indicating that the ATP level in the culture supernatant changes according to the growth phase. The extracellular ATP levels peaked at 12 to 30 nM/OD_600nm_ at 6 hours of growth that corresponds to the transition from the log phase to the stationary phase. The extracellular ATP levels then decreased as the bacterial cultures entered the stationary phase and all strains tested displayed very low extracellular ATP levels compared to those in the log phase cultures (Figure 3).

### Cytochrome bo oxidase contributes to ATP in culture supernatant

We have shown above that extracellular ATP can be detected in the culture supernatant during bacterial growth and its level peaked at the end of the log phase of growth. Next we determined if extracellular ATP is associated with cell respiration. ATP in bacteria is produced by ATP synthase powered by the proton gradient generated by the terminal oxidases [18]. *E. coli* and *Salmonella* have a variety of terminal oxidases that are active under different growth conditions. The main oxidases for the culture conditions we used (LB broth, 37°C, aerobic growth) include cytochrome bo oxidase, cytochrome bd I and II oxidases [18]. To determine if and which oxidase or oxidases contribute to the ATP detected in the culture supernatant, we obtained a panel of mutants that each contained a deletion mutation in one of the subunits encoding the terminal oxidases [19] [Coli Genetic Stock Center, Yale University]. The growth properties and ATP levels in the culture supernatant from each mutant were determined (Table 3).

All strains of terminal oxidase mutants grew normally under the assay condition, and the only exception was the cytochrome bd-I oxidase mutant ∆*cydB* that displayed a growth delay in the log phase (Table 4 and data not shown). The peak extracellular ATP level of the ∆*cydB* mutant at 6 hours of incubation was very low at 1.3 ± 2.2% of that of the parental strain. However; because of the growth defect of the ∆*cydB* mutant it was not possible to distinguish if the decreased ATP level was caused directly by the lack of the cytochrome bd I oxidase activity or indirectly by the slow growth of the ∆*cydB* mutant. Therefore the ∆*cydB* mutant was not analyzed further. In contrast to the cytochrome bd-I oxidase mutant ∆*cydB*, all mutants of the cytochrome bo oxidase and the cytochrome bd II oxidase grew normally (data not shown). The peak extracellular ATP levels in mutants lacking one of the subunits of cytochrome bo oxidase (∆*cyoA*, ∆*cyoC* and ∆*cyoD* mutants) ranged from 26.1% to 36.6% of that of the wild type level (*p* < 0.05, Student’s *t*-test). The peak ATP level from the mutant lacking cytochrome bd II oxidase (∆*appC*) was 94.8 ± 2.5% of that of the parental strain; the difference is small but is statistically significant (*p* < 0.05, Student’s *t*-test) (Table 4).

**Table 4:** **Peak ATP levels in culture supernatant of terminal oxidase mutants of *E. coli***

**Enzyme**	**Mutant**	**Growth property**	**% of the WT level**	***p*, student’s *t*-test**
Cytochrome bd-I oxidase	*∆cydB*	Defective	1.3 ± 2.2	< 0.05
Cytochrome bd-II oxidase	*∆appC*	Normal	95.0 ± 2.5	< 0.05
Cytochrome bo oxidase	*∆cyoA*	Normal	25.0 ± 3.7	< 0.05
	*∆cyoC*	Normal	36.6 ± 1.5	< 0.05
	*∆cyoD*	Normal	26.1 ± 5.4	< 0.05

Results are the average of three assays with standard deviations.

**Figure 4:**
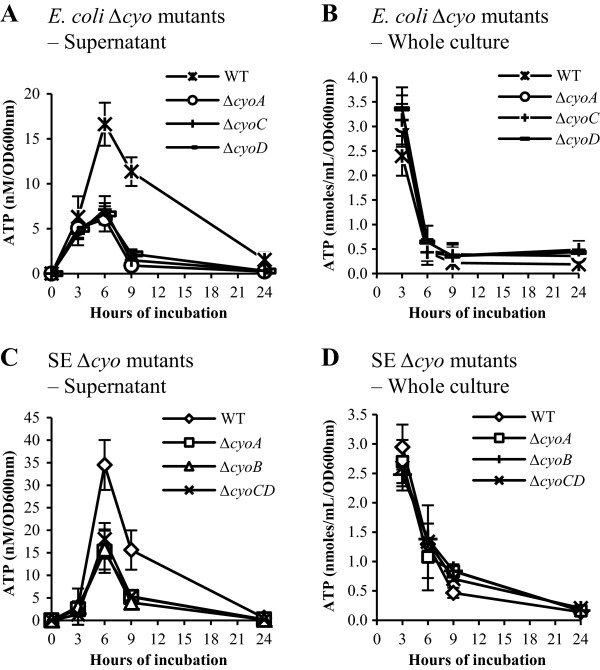
**The *∆cyo *mutants of *E. coli *BW25113 and *Salmonella *SE2472 have lower extracellular ATP levels during growth.** Overnight cultures of wild type (WT) or *∆cyo* mutants of *E. coli***(A and B)** or *Salmonella***(C and D)** were diluted 1:100 in fresh LB broth and cultured at 37°C with shaking. Aliquots were collected at various time points and ATP assays were carried out with culture supernatant or whole culture. The ATP levels in the culture supernatant **(A and C)** or whole culture **(B and D)** were normalized using OD_600nm_ and plotted against the incubation period. Results are the average of 3 experiments and error bars represent standard deviations.

The cytochrome bo oxidase mutants of *E. coli* were analyzed further to characterize the extracellular ATP levels during growth. While the extracellular ATP levels in the ∆*cyo* mutants displayed time courses similar to that of the parental strain, the peak levels were significantly lower than that observed in the parental strain (Figure 4A). These results suggest that cytochrome bo oxidase contributes to the extracellular ATP even though it had no significant influence on the growth of *E. coli* under the conditions used for the assay (LB broth, 37°C, with shaking). The cytochrome bo oxidase was then mutated in *Salmonella* to determine if the oxidase contributes to the extracellular ATP in *Salmonella* as observed in *E. coli*. Deletion mutations were generated for *cyoA*, *cyoB* and *cyoC/D*[15,16] and the mutants were assayed for their extracellular ATP levels during growth. Similar to what was observed in *E. coli*, the ∆*cyo* deletion mutants produced less extracellular ATP compared to the wild type parental strain (Figure 4C).

The decreased levels of the extracellular ATP of the ∆*cyo* mutants could be due to an overall ATP production defect in the mutants or due to a decreased release of ATP. To determine which the case is for the ∆*cyo* mutants, the ATP levels were determined in the bacterial whole culture and plotted for each mutant. As shown in Figure 4B and D, the ∆*cyo* mutants of both *E. coli* and *Salmonella* contained comparable quantities of ATP in the bacterial whole cultures. Therefore, the decreased levels of extracellular ATP from the cytochrome bo oxidase mutants of *E. coli* and *Salmonella* were not due to any obvious ATP synthesis deficiency.

### Bacterial cultures deplete ATP in the culture medium

**Figure 5:**
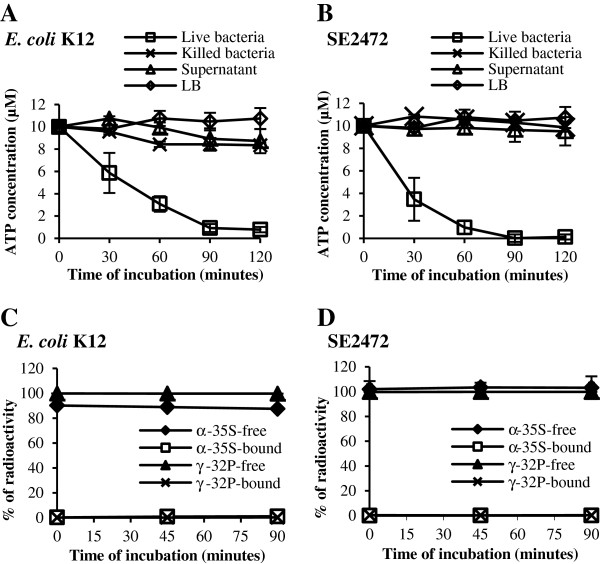
**Live bacteria deplete ATP in the culture medium. (A)** and **(B)** Live or heat – killed *E. coli* K12 **(A)** or *Salmonella* SE2472 **(B)** were spun down and incubated at 37°C in fresh LB supplemented with 10 μM ATP. Culture supernatant from live bacteria was supplemented with ATP to 10 μM. ATP depletion by bacteria cells or culture supernatant was measured by the residual ATP level in culture medium after various culture periods of incubation at 37°C. The residual ATP levels were plotted against the incubation period. **(C)** and **(D)** Free and cell-associated ATP in *E. coli***(C)** or *Salmonella***(D)** culture incubated with S^35^-α-ATP or P^32^-γ-ATP. The relative levels of radioactivity in culture supernatant and bacterial cells were determined and plotted against the incubation period. Each experiment was performed three times and results are from a representative experiment.

As shown in Figures 3 and 4 the presence of extracellular ATP in the culture supernatant of *E. coli* and *Salmonella* peaked at the late log phase. To investigate why the extracellular ATP level decreases as bacteria enter into stationary phase of growth, we measured if *Salmonella* and *E. coli* cultures deplete ATP in the culture medium. Overnight cultures were spun down and the culture supernatant was removed. Bacteria were then resuspended in fresh LB supplemented with 10 μM ATP and the ATP level in the culture medium was measured at various time points of incubation. The ATP level decreased rapidly in culture medium incubated with either *E. coli* or *Salmonella* (Figure 5A and B). The ATP depletion requires live bacteria as heat-killed bacteria, culture supernatant or LB broth depleted little of supplemented ATP (Figure 5A and B). Over 2 h of incubation live bacteria depleted approximately 10 μM ATP, which was several magnitudes higher than the usual 20–100 nM of extracellular ATP detected in *E. coli* or *Salmonella* cultures (Figures 2, 3 and 4). These results suggest that the capacity of ATP depletion by *E. coli* and *Salmonella* far exceeds the peak level of the extracellular ATP detected in bacterial culture supernatant.

Since bacterial cells instead of culture supernatant deplete ATP (Figure 5A and B), we reasoned that the reduction of ATP level in the culture supernatant could be due to hydrolysis or degradation of ATP at the bacterial cell surface. Alternatively, ATP level can become lower due to an uptake by bacteria although no ATP transporter or uptake system has been reported in bacteria. To explore the fate of the extracellular ATP, we incubated bacteria with ^35^S -α-ATP and quantified the radioactivity in the culture supernatant and bacterial pellet. ATP transported back into bacteria should be detected by cell-associated radioactivity whether it remains as ATP or is hydrolyzed subsequently into ADP or AMP. Stationary phase cultures of *Salmonella* and *E. coli* were spun down and resuspended in fresh LB broth supplemented with ^32^S-α-ATP. After various periods of incubation, bacteria were spun down, washed, and the radioactivity was measured in the culture supernatant or in the bacterial cell pellet. Virtually all radioactivity remained in the culture supernatant and very little radioactivity was detected in bacterial cell pellet of *Salmonella* or *E. coli* (Figure 5C and D).

We next tested if the extracellular ATP was used in kinase reactions to phosphorylate proteins and other cell surface components. ATP depletion assay was carried out using ^32^P -γ-ATP as described above for ^32^S-α-ATP. Quantitation of radioactivity in the culture supernatant and bacterial pellet showed that radioactivity was present almost exclusively in the culture supernatant (Figure 5C and D). This suggests that ATP was most likely hydrolyzed or degraded by bacteria on their surface and was not transported into bacteria or used for phosphorylating bacterial components.

### Extracellular ATP enhanced stationary survival of *E. coli* and *Salmonella*

**Figure 6:**
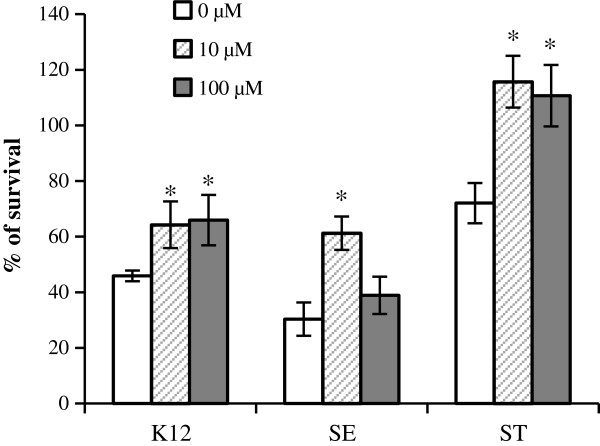
**ATP supplementation increases the stationary survival of bacteria.***E. coli* K12, *Salmonella enterica* Serovar Enteritidis (SE) or *Salmonella enterica* Serovar Typhimurium (ST) was cultured in M9 minimal medium or M9 minimal medium supplemented with 10 μM or 100 μM of ATP. The rate of survival was determined by comparing bacterial CFU/mL after 7 days of incubation to that after 1 day of incubation. The experiment was performed three times and results are from a representative experiment performed in triplicate. Error bars represent standard deviation. * *p* < 0.05, Student’s *t*-test.

The presence of the extracellular ATP in bacterial cultures was unexpected since it likely represents a loss of the valuable small molecule to bacteria. The extracellular ATP could be an unavoidable cost to bacterial respiration or could be beneficial to bacteria in some aspects. We next determined if the extracellular ATP has any impact on the growth and survival characteristics of bacteria. Since extracellular ATP level was found to decrease during the stationary phase of growth (Figure 3), we determined if the extracellular ATP is beneficial to bacteria at stationary phase and if ATP supplement could enhance the bacterial survival. *Salmonella* and *E. coli* were cultured for 7 days and exogenous ATP was added to the cultures. We chose to use 10 μM or 100 μM to supplement bacterial culture since the ATP depletion assays showed that *Salmonella* and *E. coli* depletes ATP at approximately 5 μM/hr (Figure 5A and B) and high concentrations of ATP would allow ATP level in the bacterial cultures to stay elevated for an extended period of time. Survival of bacteria was determined by the ratio of bacterial CFU/mL after 7 days of incubation to that after 1 day of incubation (Figure 6). Our results showed that an ATP supplement increased the survival of the bacterial strains tested. The dosage response varied in different strains. *Salmonella* responded best to 10 μM ATP, while *E. coli* responded equally well to 10 μM and 100 μM ATP. The results suggest that extracellular ATP can affect bacterial survival (Figure 6).

### Extracellular ATP was detected in several Gram-negative and Gram-positive bacterial species

In addition to Gram-negative bacterial species *E. coli* and *Salmonella*, other bacterial species were tested for the presence of ATP in the culture medium to determine if the phenomenon is limited to Enterobacteriaceae or is present in more bacterial families such as *Acinetobacter*, *Klebsiella*, *Pseudomonas* and *Staphylococcus*. Clinical isolates of various human pathogenic bacterial species were tested for the presence of ATP in culture medium during their growth *in vitro* and the ATP levels in the culture supernatant were determined. The peak values of the ATP concentration in the culture medium and the incubation time when the ATP levels peaked are listed in Table 5. ATP was detected in the culture supernatant of all bacterial strains tested. Although the levels and peak time points varied from strain to strain, all bacterial strains displayed the presence of growth phase dependent ATP in the culture supernatant (Table 5). This result suggests that the presence of extracellular ATP is not restricted to Enterobacteriaceae and instead can be detected in many bacterial families. For most bacterial strains the ATP levels detected were comparable to those observed in *Salmonella* and *E. coli* which peaked around 10 – 30 nM/OD_600nm_ (Figures 3 and 4). Some bacterial strains, however, displayed much higher or lower ATP levels. For example, a clinical isolate of *Acinetobacter junii* (AJ4970) had a peak extracellular ATP level of > 250 nM/OD_600nm_, several fold higher than the peak concentrations observed in most bacterial strains (Table 5). In contrast a clinical isolate of *Klebsiella pneumoniae* had a low peak ATP level of approximately 1 nM/OD_600nm_ (Table 5). The extracellular ATP did not appear to display a species – specific pattern and strains from the same bacterial species could have very different peak ATP levels (e.g. AJ4970 at 255.2 ± 56.8 nM/OD_600nm_ vs. AJ4978 at 17.0 ± 1.1 nM/OD_600nm_), suggesting that extracellular ATP is a common phenomenon to many bacterial species while the dynamics of ATP release is different in each bacterial strain.

**Table 5:** Extracellular ATP from various bacterial species

**Strain**	**Species**	**Peak hour**	**Peak level (nM/OD)**
AJ4970	*Acinetobacter junii*	6	255.2 ± 56.8
AJ4978	*Acinetobacter junii*	6	17.0 ± 1.1
PA292	*Pseudomonas aeruginosa*	6	25.5 ± 1.1
PA4553	*Pseudomonas aeruginosa*	3	20.5 ± 0.6
KP7690	*Klebsiella pneumoniae*	9	9.3 ± 0.5
KP2320	*Klebsiella pneumoniae*	9	1.0 ± 0.0
KO76	*Klebsiella oxytoca*	3	31.1 ± 4.0
SA25923	*Staphylococus aureus*	6	21.4 ± 3.5
MRSA43300	*Staphylococus aureus*	6	19.3 ± 1.3

Results are the average of three assays with standard deviations.

**Figure 7:**
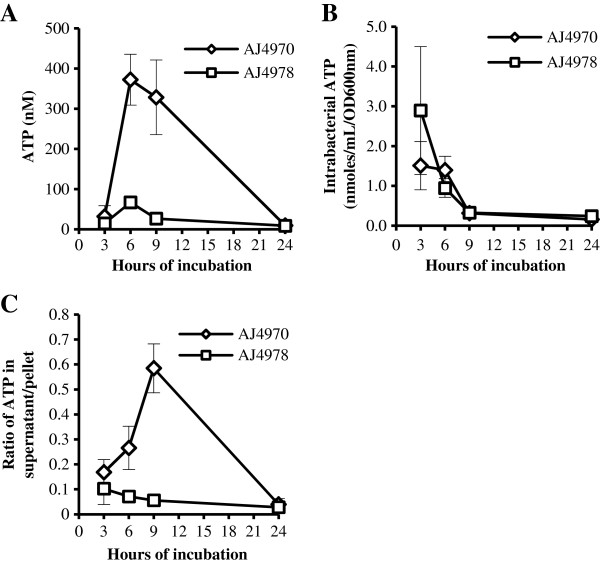
**ATP levels in the cultures of *Acinetobacter junii*.** Overnight cultures of two clinical isolates of *Acinetobacter junii* AJ4970 and AJ4978 were diluted 1:100 in fresh LB broth and cultured at 37°C with shaking. Aliquots were collected at various time points and ATP levels in the culture supernatant and the bacterial pellet were determined. **(A)** ATP levels in the culture supernatant. ATP concentrations were determined and plotted against the incubation period. **(B)** ATP levels in the bacterial pellet. Total ATP levels in the bacterial pellet were normalized against OD_600nm_ of each culture and plotted against the incubation time period. **(C)** Ratio of quantity of ATP in the culture supernatant to that of the bacterial cells. *Acinetobacter junii* cultures were spun down and separated into culture supernatant and cell pellet. ATP levels in each fraction were determined. The ratio of ATP from supernatant to that of bacterial cells from the same volumes of cultures was plotted against the incubation period. Results are the average of 4 experiments and error bars represent standard deviations.

The ATP levels of two isolates of *Acinetobacter junii* AJ4970 and AJ4978 were analyzed in more details to compare the quantity of ATP in the culture supernatant to that in bacterial cells. Overnight culture of AJ4970 or AJ4978 was diluted 1:100 in fresh LB broth and cultured at 37°C with shaking. Aliquots were collected at various time points and the ATP levels in the culture supernatant and bacterial pellet were determined (Figure 7A and B). The ratio of total ATP in the supernatant to that in the bacterial pellet from the same volume of bacterial culture was also determined (Figure 7C). The ATP level in the culture supernatant of AJ4970 reached a peak level of over 300 nM at 6 hours of incubation (Figure 7A) and the ratio of ATP in the culture supernatant to that in the pellet (total ATP in supernatant/total ATP in the pellet) peaked at 0.58 at 9 hours of incubation (Figure 7C). By comparison AJ4978 displayed much lower ATP levels in the culture supernatant as well as lower supernatant/pellet ratios of ATP (Figure 7A and C). The ATP levels in the bacterial cells were comparable in AJ4970 and AJ4978, except that AJ4978 had a higher intracellular ATP level at 3 hours of incubation (Figure 7B).

## Discussion

We report here that ATP can be detected in the culture supernatant of a wide variety of bacterial species including both Gram-positive and Gram-negative bacteria of laboratory and clinical strains (Figure 2 and Table 5). The concentrations of extracellular ATP (from several nanomolar to several hundred nanomolar) were much lower than the 1–5 mM reported for intracellular ATP [6-9], and total extracellular ATP represents up to 3 to 5% of that in bacterial culture (Figure 4). One noticeable exception is *Acinetobacter junii* AJ4970 that had ratios of extracellular to intracellular ATP > 0.5 (Figure 7C), suggesting that a significant portion of total ATP was present in the culture supernatant of this bacterial strain.

The extracellular ATP is unlikely an artifact due to any contamination of culture supernatant by bacterial cells since filtration did not reduce the ATP level (Figure 1). However, we have yet to establish the mechanism of how ATP was released into the culture medium. The simplest explanation is that ATP was released from dead and lysed bacteria. This explanation is plausible for low extracellular ATP levels when total extracellular ATP is less than 5% of the intracellular ATP levels; however, it cannot explain the high extracellular ATP levels observed with AJ4970 which has comparable quantities of extracellular ATP compared to the intracellular ATP (Figure 7C). In addition we have shown that live bacteria of both *E. coli* and *Salmonella* (but not dead bacteria or culture supernatant) are able to actively deplete ATP at approximately 5 μM/hr or 83 nM/min (Figure 5A and B) – a very high rate compared to the peak extracellular ATP concentration of 15 nM to 35 nM/OD_600nm_ in *E. coli* and *Salmonella* cultures (Figure 4). Thus the quantity of ATP released into culture supernatant is likely to be much higher than that detected in the supernatant. Genetic analysis showed that ATP release is linked to cytochrome bo oxidases and thus argues against the bacterial cell death and lysis as the sole source of the extracellular ATP (Figure 4).

We show here that the extracellular ATP levels are regulated by growth phase in all bacterial species tested (Figure 3 and Table 5). They peaked at the late log to early stationary phase of growth for most strains and decreased to much lower or undetectable levels by 24 hours of growth. The growth phase – dependent presence of extracellular ATP suggests a dynamic process of ATP release and depletion, and the observed level of ATP in the culture supernatant is most likely the combined effect of the two processes. Live *E. coli* and *Salmonella* (but not dead bacteria or culture supernatant) appear to actively deplete extracellular ATP and the depletion was not due to uptake (Figure 5). Either α-labeled or γ-labeled phosphate on supplemental ATP remained in the culture medium, suggesting that the extracellular ATP was hydrolysed or degraded at the bacterial surface (Figure 5).

There have been a few reports on the extracellular ATP from bacteria [1,9,10]. Iwase et al. reported the detection of ATP in the culture supernatant of *Enterococcus* species, but not strains of *E. coli* or *Staphylococcus aureus* (Iwase, 2010 #195). A possible reason for the discrepancy between their results and ours is that they used overnight cultures which had very low ATP levels in our study as well, while cultures at late log and early stationary phases had much higher extracellular ATP levels (Figures 3 and 4). Another report by Ivanova et. al reported the presence of extracellular ATP from cultures of *Sulfitobacter*, *Staleya* and *Marinobacter* at 190 μM to 1.9 mM. These levels approach those reported for intracellular ATP of 1 – 3 mM and are much higher than we observed. If those levels are accurate it would suggest that the total quantity of extracellular ATP far exceeds that of intracellular ATP since the volume of cell culture medium is at least several hundred times higher than that of bacterial cells. We do not know if the differences between results by Ivanova et al. and our results were due to the different bacterial species used or to technical reasons. After we finished the experiments reported here and were preparing this manuscript, Hironaka et al. reported a follow-up study to their previous report that ATP is secreted by gut commensal bacteria [11]. In the new report, they demonstrated that ATP can be detected in the culture supernatant of log phase cultures of *E. coli*, *Pseudomonas aeruginosa* and *Staphylococcus aureus* but not the stationary cultures, in agreement with our observations reported here [11]. They also reported that glycolysis is essential for ATP secretion which supports our notion that cytochrome bo oxidase and respiration are important for ATP release (Figure 4).

Reports in recent years have shown that eukaryotic cells can release ATP without lysis through exocytosis of ATP-containing granules, plasma membrane carriers or large conductance channels [2,3,20,21]. Cells of innate immunity such as dendritic cells and macrophages sense ATP as a danger signal through purinergic receptors of P1 and P2 family and initiate a pro-inflammatory response [2,3,20]. It is possible that ATP serves as a signaling molecule to regulate both eukaryotic cells and bacteria in the bacteria – host interaction. Atarashi et al. reported that T_H_17 T-helper cells in the intestinal lamina propria are induced by intestinal ATP [1]. Germ – free mice were shown to have lower luminal concentration of ATP and fewer numbers of T_H_17 cells, and the number of T_H_17 cells increased by systemic or rectal administration of ATP [1]. The source of intestinal ATP was not identified but was presumably commensal bacteria, which is supported by our findings that many bacterial species release ATP. A recent report by Lee and Groisman demonstrated that ATP regulates *Salmonella* virulence gene *mtgC*[4]. We have shown that ATP supplement of 10 μM or 100 μM increased the survival of *Salmonella* at the stationary phase (Figure 6). The ATP supplement of 10 μM or 100 μM was much higher than the observed extracellular ATP concentrations in bacterial cultures (~ 30 to 50 nM), but the concentration of the ATP supplement was still much lower than the intracellular ATP concentrations of 1 mM – 10 mM reported for eukaryotic cells [22-24]. An intracellular pathogen such as *Salmonella* is likely to be exposed to ATP inside host cells and our results suggest that *Salmonella* is capable of utilizing ATP to increase its survival, possibly by using extracellular ATP as a nutrient and/or a signaling molecule. Regardless of the exact role of extracellular ATP, intracellular pathogens such as *Salmonella* would have access to host ATP inside host cells and the ability to use extracellular ATP should be beneficial to the intracellular pathogens.

We have detected extracellular ATP from a variety of bacterial species, suggesting that extracellular ATP is not limited to any particular bacterial species. The biological purpose of ATP release is yet to be determined. Since bacteria likely exist as communities in their natural state, a possible role for the extracellular ATP is to function as a nutrient or a signaling molecule in the bacterial communities. It can be a signal in quorum sensing as it changes with bacterial density (Figures 3 and 7). Though less likely, ATP release could be an altruistic action of individual bacterium that facilitates the formation and survival of bacterial communities. Indeed our results show that exogenous ATP increased the stationary survival of *E. coli* and *Salmonella* (Figure 6). It is possible that ATP released from some members of the bacterial communities may supply energy to other members and hence help the communities thrive. The role of extracellular ATP and the mechanisms of ATP release need further characterization; nevertheless the current study indicates that ATP is present extracellularly and may have additional functions in bacterial physiology in addition to its role as an energy supplier.

## Conclusions

We have detected extracellular ATP in the culture supernatant of several Gram – positive and Gram – negative bacterial species. The extracellular ATP level in all bacterial strains tested was growth-phase dependent with the highest level at the late log phase of growth. The presence of extracellular ATP and the dynamic changes in its level suggest that ATP may have important functions extracellularly in addition to its long-established roles intracellularly.

## Competing interests

The authors declare that they have no competing interests.

## Authors’ contributions

RM participated in the study design, performed the experiments and helped to draft the manuscript. HT, CC, HG and KH performed the experiments. SL conceived of the study, participated in the study design, performed the experiments, performed the statistical analysis and drafted the manuscript. All authors read and approved the final manuscript.
